# Improved survival prediction and comparison of prognostic models for patients with hepatocellular carcinoma treated with sorafenib

**DOI:** 10.1111/liv.14270

**Published:** 2019-11-18

**Authors:** Tim A. Labeur, Sarah Berhane, Julien Edeline, Jean‐Frederic Blanc, Dominik Bettinger, Tim Meyer, Jeroen L. A. Van Vugt, David W. G. Ten Cate, Robert A. De Man, Ferry A. L. M. Eskens, Alessandro Cucchetti, Laura J. Bonnett, Otto M. Van Delden, Heinz‐Josef Klümpen, R. Bart Takkenberg, Philip J. Johnson

**Affiliations:** ^1^ Cancer Center Amsterdam Amsterdam The Netherlands; ^2^ Department of Medical Oncology Amsterdam University Medical Centers University of Amsterdam Amsterdam The Netherlands; ^3^ Department of Gastroenterology and Hepatology Amsterdam University Medical Centers University of Amsterdam Amsterdam The Netherlands; ^4^ Department of Radiology and Nuclear Medicine Amsterdam University Medical Centers University of Amsterdam Amsterdam The Netherlands; ^5^ Department of Biostatistics University of Liverpool Liverpool UK; ^6^ Department of Oncology Centre Eugène Marquis Rennes France; ^7^ Department of Hepatology CHU Hôpital Saint André Bordeaux France; ^8^ Department of Medicine II Medical Center University of Freiburg Faculty of Medicine University of Freiburg Freiburg Germany; ^9^ UCL Cancer Institute University College London London UK; ^10^ Department of Surgery Erasmus MC University Medical Center Rotterdam The Netherlands; ^11^ Department of Gastroenterology and Hepatology Erasmus MC University Medical Center Rotterdam The Netherlands; ^12^ Department of Medical Oncology Erasmus MC University Medical Center Rotterdam The Netherlands; ^13^ Department of Medical and Surgical Sciences Alma Mater Studiorum University of Bologna Bologna Italy; ^14^ Department of Molecular and Clinical Cancer Medicine University of Liverpool Liverpool UK

**Keywords:** hepatocellular carcinoma, model, prediction, prognosis, sorafenib, survival

## Abstract

**Background:**

The ‘Prediction Of Survival in Advanced Sorafenib‐treated HCC’ (PROSASH) model addressed the heterogeneous survival of patients with hepatocellular carcinoma (HCC) treated with sorafenib in clinical trials but requires validation in daily clinical practice. This study aimed to validate, compare and optimize this model for survival prediction.

**Methods:**

Patients treated with sorafenib for HCC at five tertiary European centres were retrospectively staged according to the PROSASH model. In addition, the optimized PROSASH‐II model was developed using the data of four centres (training set) and tested in an independent dataset. These models for overall survival (OS) were then compared with existing prognostic models.

**Results:**

The PROSASH model was validated in 445 patients, showing clear differences between the four risk groups (OS 16.9‐4.6 months). A total of 920 patients (n = 615 in training set, n = 305 in validation set) were available to develop PROSASH‐II. This optimized model incorporated fewer and less subjective parameters: the serum albumin, bilirubin and alpha‐foetoprotein, and macrovascular invasion, extrahepatic spread and largest tumour size on imaging. Both PROSASH and PROSASH‐II showed improved discrimination (C‐index 0.62 and 0.63, respectively) compared with existing prognostic scores (C‐index ≤0.59).

**Conclusions:**

In HCC patients treated with sorafenib, individualized prediction of survival and risk group stratification using baseline prognostic and predictive parameters with the PROSASH model was validated. The refined PROSASH‐II model performed at least as good with fewer and more objective parameters. PROSASH‐II can be used as a tool for tailored treatment of HCC in daily practice and to define pre‐planned subgroups for future studies.

**Abbreviations**
95% CI: 95% confidence interval
AIC: akaike information criterion
ALBI: albumin‐bilirubin
APF: alpha‐foetoprotein
AST: asparatate transaminase
BCLC: barcelona clinic liver cancer
CT: computed tomography
DCP: des‐gamma‐carboxyprothrombin
ECOG PS: Eastern Cooperative Oncology Group performance status
HAP: hepatoma arterial‐embolization prognostic
HCC: hepatocellular carcinoma
JIS: Japan Integrated Staging (JIS) score
LR: likelihood ratio
MRI: magnetic resonance imaging
NLR: neutrophil‐to‐lymphocyte ratio
OS: overall survival
PROSASH: Prediction Of Survival in Advanced Sorafenib‐treated Hepatocellular carcinoma
SAP: sorafenib advanced hepatocellular carcinoma prognostic
SD: standard deviation
SIRT: selective internal radiation therapy

**Key Points**

- Patients with incurable liver cancer (hepatocellular carcinoma) can be treated with sorafenib to expand their life expectancy, but the prognosis with this drug varies between patients.
- In this large international study, we tested and further improved a statistical method that allows clinicians to estimate the survival chances of an individual patient.
- This facilitates the communication with the patient when considering this treatment and will help further research to find better drugs.

## 1 INTRODUCTION

Hepatocellular carcinoma (HCC) is the most common primary liver cancer and the second leading cause of cancer‐related death worldwide.^1^ Most patients with HCC present with, or eventually progress to, advanced stage disease which bears a poor prognosis. Sorafenib, a multikinase inhibitor, was the first treatment to show a survival benefit in patients with advanced stage HCC. In two randomized‐controlled trials, sorafenib improved the median overall survival (OS) by 2‐3 months compared with placebo.^2^, ^3^ Since then, sorafenib has been the standard treatment for patients with advanced stage HCC who are ineligible for loco‐regional treatment and have preserved (Child‐Pugh A) liver function.

However, there is significant heterogeneity in outcomes in patient treated with sorafenib with an OS ranging from <3 months to 2‐3 years.^2^, ^3^, ^4^ This indicates that the survival benefit offered by sorafenib varies between individual patients. Select subgroups may have similar or more benefit from alternative options such as lenvatinib,^5^ best supportive care or clinical trials.

The variety in survival is inadequately captured by the currently available staging systems (ie Barcelona Clinic Liver Cancer [BCLC]). Therefore, guidelines have recommended exploration of further stratification of patients with intermediate (BCLC‐B) and advanced stage HCC (BCLC‐C).^6^ Previous studies have identified markers of liver function (ie albumin, bilirubin), clinical parameters (ie performance status, body composition) and tumour characteristics (ie alpha‐foetoprotein [AFP], macrovascular invasion, tumour extent) that may aid in prognostic stratification prior to sorafenib treatment.^7^, ^8^, ^9^, ^10^, ^11^, ^12^, ^13^, ^14^, ^15^ Predictive factors, that is, those associated with improved survival benefit over placebo, included absence of extrahepatic spread, presence of hepatitis C virus and a low neutrophil‐to‐lymphocyte ratio (NLR).^16^ Based on the combination of baseline factors, several scoring systems have been proposed for survival stratification of patients with advanced HCC treated with sorafenib.^17^, ^18^, ^19^, ^20^ Limitations of these models include the use of factors that either have a degree of subjectivity (ie infiltrative tumour growth, ascites) or are not commonly available (ie Des‐gamma‐carboxyprothrombin [DCP]). A recently proposed model, the ‘Prediction Of Survival in Advanced Sorafenib‐treated HCC’ (PROSASH), provided individualized survival prediction with excellent risk group discrimination based on nine parameters (age, macrovascular invasion, extrahepatic spread, performance status, disease aetiology, albumin, creatinine, aspartate transaminase (AST) and AFP).^21^ The PROSASH model was built and validated on the data from patients treated with sorafenib in two clinical trials,^22^, ^23^ but has not yet been validated in patients treated in routine clinical practice. Multiple studies in various tumour types have underlined the limited applicability of data from the strictly selected and homogeneous patients treated in clinical trials to the more heterogeneous population in routine clinical practice.^24^, ^25^, ^26^, ^27^, ^28^ Moreover, the PROSASH model has not yet been compared with the currently existing prognostic scores (BCLC, Child‐Pugh). Consequently, it remains unknown whether this new model outperforms the existing models and whether risk stratification of sorafenib‐treated patients might be further refined using data from ‘real‐life’ patients.

Therefore, this study aimed to (1) validate the PROSASH model in HCC patients treated with sorafenib in daily clinical practice and (2) improve the PROSASH based on patients treated in clinical practice. Subsequently, PROSASH, the improved model (PROSASH‐II) and existing prognostic models were compared to determine the utility for clinicians to predict the survival of these patients.

## 2 MATERIALS AND METHODS

### 2.1 Study population

Patients with HCC treated with sorafenib were recruited consecutively at five tertiary European centres with specialist multidisciplinary services for HCC management: Bordeaux (n = 306) and Rennes (n = 129), France; Freiburg (n = 183), Germany; Amsterdam (n = 156) and Rotterdam (n = 167), the Netherlands. The data were collected after obtaining the relevant authorization from the institutional review boards and this retrospective study was performed under ethically approved protocols (REC reference 12/LO/1088 and W17_420#17.488). Patients were diagnosed with HCC by histological or radiological criteria in accordance with international guidelines.^6^, ^29^ Exclusion criteria included patients receiving combination treatments (ie selective internal radiation therapy [SIRT] with sorafenib) or those with an Eastern Cooperative Oncology Group performance status (ECOG PS) >2. Patients received sorafenib with a target dose of 400 mg BID, with toxicity‐adjusted dosing and patient management according to the local practice.

### 2.2 Data collection and outcomes

Commonly available clinical, imaging and serum variables prior to sorafenib treatment were collected by members of the research team. Imaging parameters were obtained from the most recent radiological imaging prior to first dose of sorafenib. Radiological staging included a multiphasic contrast‐enhanced computed tomography (CT) or dynamic magnetic resonance imaging (MRI). The main outcome measure, OS, was defined from the date of start of treatment to date of death or censored on the date of last follow‐up.

Patients were staged according to the PROSASH model.^21^ To assess whether improved prediction may be possible using data from daily practice, a new model was built and validated (PROSASH‐II, detailed below). The utility of both models was compared with existing prognostic scores that could be assessed in the dataset, including the BCLC staging system, Child‐Pugh classification, albumin‐bilirubin (ALBI) grade,^30^ Japan Integrated Staging (JIS) score,^31^ hepatoma arterial‐embolization prognostic (HAP)^32^ and the Sorafenib Advanced HCC Prognostic (SAP) score.^18^ With the exception of BCLC stage and Child‐Pugh classification, which are commonly used in daily practice and were coded by the individual investigators, all prognostic scores were calculated using the raw data.

### 2.3 Statistical methods

Continuous variables were described as means with standard deviation (SD) or medians with interquartile range in case of highly skewed distributions. Categorical variables were described as absolute and relative frequencies. The Kaplan‐Meier method was used to generate and compare survival curves, and to estimate median OS with 95% confidence interval (95% CI). For all analyses, a two‐tailed *P* < .05 was considered statistically significant. Statistical analysis was performed using SPSS Statistics for Windows Version 24.0 (IBM Corp) and STATA/SE 14.1 (StataCorp).

#### 2.3.1 Model building, testing and external validation

For the building of a prognostic model from patients treated in daily practice, the data of four centres were clustered into a training dataset and the largest independent dataset (Bordeaux) was used as an external validation set. Baseline variables that were considered clinically relevant and available in both datasets were included in the model building process (Table S1). Highly skewed variables were log‐transformed. BCLC stage and Child‐Pugh grade were excluded from the model building process owing to multicollinearity with factors used in these scoring systems. Multiple imputations (10x) using chained equations were performed to account for missing key parameters that were missing at random in the training dataset.^33^, ^34^ Model performance, derived coefficients and *P* values of imputed data were compared with complete case data.

In the training set, the association between OS and baseline variables was assessed in an exploratory univariable and subsequent multivariable flexible parametric survival analysis.^35^, ^36^, ^37^ The advantages of a flexible parametric analysis over the more commonly used Cox proportional hazard analysis were previously described.^21^, ^37^ Risk factors were reported with hazard ratio (HR) and corresponding *P* values. The multivariable model was built using a stepwise forward selection procedure of variables significant at the 5% level. The model was reported according to the TRIPOD guidelines^38^ as well as tested, optimized and validated using the methods described by Royston and Altman.^39^ Any time‐dependent effects and potential proportional hazard violations by variables in the model were examined using the likelihood ratio (LR) test.^37^ The LR test was also used to optimize the degrees of freedom (number of knots) for the restricted cubic spline function.^37^ Lastly, Martingale residuals were plotted against continuous variables to check the functional form and non‐linearity.

A linear predictor was derived using the coefficients of the model variables. Four risk groups were generated by applying the previously suggested cut‐offs at the 16th, 50th and 84th centiles of the training set's linear predictor.^39^ The model, including the linear predictor and the centile‐based risk group stratification, was applied to the external validation set.

The calibration of survival prediction was visually assessed by comparing the similarity between the observed and predicted survival curves in both the training and validation set. The observed and predicted survival‐percentage at 12 months were also compared. Model discrimination was visually inspected by examining the separation survival curves of the four risk groups. In addition, survival rates between the risk groups were compared using HRs or log‐rank test and the accompanying *P* values. Lastly, subgroup analyses of the new model were performed in patients with Child‐Pugh A or Child‐Pugh B because current guidelines recommend selecting patients with Child‐Pugh A patients only.^6^, ^29^

#### 2.3.2 Model comparison

The PROSASH model incorporates the variable ‘aspartate transaminase (AST)’ which was not available in the Rennes (training) and Bordeaux (validation) datasets. Therefore, model comparisons were performed in three subgroups of patients:

1. The imputed training dataset,
2. The external validation set, with complete data for all prognostic models except for the PROSASH model and.
3. Patients with complete data for all prognostic scores.

For each prognostic model, the utility and discriminative performance was quantified using the Akaike Information Criterion (AIC) Harrell's C‐index and Royston‐Sauerbrei's R^2^_D_ 40, 41 A lower AIC indicates a better goodness of fit, whereas a higher Harrell's C‐index indicates a larger proportion of patient pairs has agreement between the survival prediction and observed survival outcome in terms of rank. A higher R^2^_D_ reflects a better explained variation on the log relative hazard scale. Most prognostic models consist of a linear predictor or point‐based system with a risk group categorization which can lead to loss of information (ie ALBI‐score and ALBI grade 1, 2 and 3). To assess the difference, the performance of each model as a linear predictor or points and risk groups was assessed. Because of lacking data, the number of Child‐Pugh points could not be calculated, thus only the Child‐Pugh classes (A, B and C) were assessed.

## 3 RESULTS

### 3.1 Study population

In total, 941 patients who received sorafenib for advanced HCC between February 2003 and December 2016 were identified for this study. Of these, 21 patients (2%) were excluded because they received a combination of sorafenib with loco‐regional treatment (n = 20) or due an ECOG PS >2 (n = 1). Subsequently, 920 patients were included in this study, of whom 615 (67%) patients were included in the training cohort and 305 patients (33%) in the external validation cohort. The baseline characteristics of both cohorts are summarized in Table 1. Both cohorts had similar baseline features except that in the external validation cohort, more patients had ECOG PS 0 (65% vs 45%, *P* < .001) and alcohol‐induced liver disease was more common (64% vs 35%, *P* < .001) compared with the training cohort, respectively. The median OS was 8.3 months (95% CI 7.6‐9.2) in all patients. There was no statistically significant difference in survival between the training and validation cohort (HR 1.05, 95% 0.91‐1.21, *P* = .128; Figure S1).

**Table 1:** Baseline characteristics

Variables	Entire cohort	Training‐set	External validation	*P* value
	n = 920	n = 615	n = 305	
Demographics				
Age, y (SD)	65 (10.5)	64 (10.8)	66 (9.5)	.003
Male sex (%)	787 (86)	512 (83)	275 (90)	.005
Liver disease				
Aetiology (%, multiple possible)				
HBV	94 (10)	77 (13)	17 (6)	.001
HCV	153 (17)	86 (14)	67 (22)	.002
Alcohol	407 (44)	213 (35)	194 (64)	<.001
Unknown/Other	407 (44)	263 (43)	64 (21)	<.001
Child‐Pugh class (%)				
A	747 (85)	507 (87)	240 (79)	<.001
B	133 (15)	73 (13)	60 (20)	
C	4 (<1)	0 (0)	4 (1)	
Tumour parameters				
ECOG PS (%)				
0	477 (52)	279 (45)	198 (65)	<.001
1	388 (42)	294 (48)	94 (31)	
2	55 (6)	42 (7)	13 (4)	
Number of liver lesions (%)				
1	229 (25)	135 (22)	94 (32)	<.001
2‐3	205 (23)	169 (28)	36 (12)	
>3	468 (52)	>3 (50)	163 (56)	
Largest tumour size, mm (IQR)	65 (37‐100)	65 (37‐100)	64 (36‐100)	.593
Macrovascular invasion (%)	348 (38)	223 (36)	125 (41)	.170
Extrahepatic spread (%)	418 (46)	305 (50)	113 (37)	<.001
BCLC stage (%)				
A	9 (1)	5 (1)	4 (1)	.032
B	220 (24)	155 (25)	65 (21)	
C	684 (74)	453 (74)	231 (76)	
D	6 (<1)	1 (<1)	5 (2)	
Prior treatments (%)				
Yes, received prior treatment	467 (51)	308 (50)	159 (52)	.558
No, sorafenib was first treatment	453 (49)	307 (50)	146 (48)	
Serum tests				
AFP, ng/mL (IQR)	141 (8‐2574)	127 (10‐2005)	184 (7‐4500)	.239
Albumin, g/L (SD)	37 (5.7)	38 (5.3)	35 (5.8)	<.001
Bilirubin, µmol/L (IQR)	15 (10‐24)	15 (10‐22)	17 (12‐28)	<.001
AST, U/L (IQR)	67 (107)	67 (107)	N/A	N/A
Creatinine, µmol/l (IQR)	73 (61‐88)	75 (62‐90)	69 (58‐81)	<.001
Survival outcomes				
Death (%)	832 (90)	559 (91)	273 (90)	.501
Overall Survival, months (95% CI)	8.3 (7.6‐9.2)	8.9 (8.0‐9.8)	7.7 (6.8‐8.8)	.534

Abbreviations: 95% CI, 95% confidence interval; AFP, Alpha‐Foetoprotein; AST, aspartate transaminase; BCLC, Barcelona Clinic Liver Cancer; ECOG PS, Eastern Cooperative Oncology Group performance status; HBV, hepatitis B virus; HCV, hepatitis C virus; IQR, interquartile range; SD, standard deviation.

### 3.2 Validation of the PROSASH model in routine clinical practice

**Figure 1:**
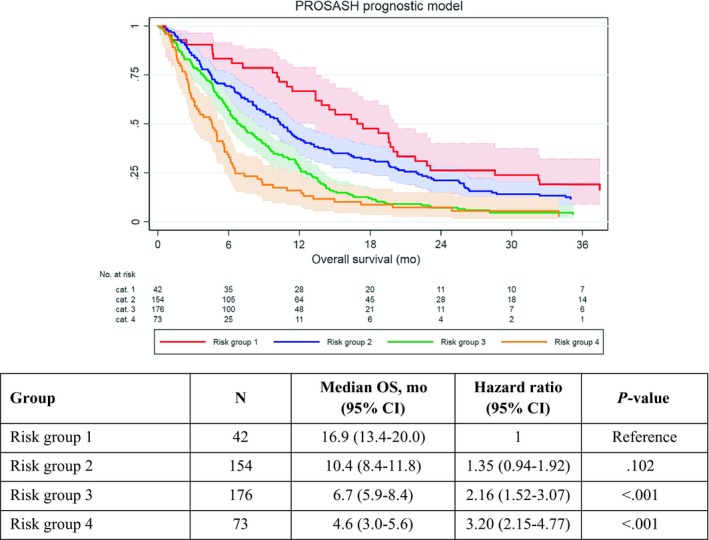
Overall survival according to the PROSASH risk groups with 95% confidence intervals

The PROSASH model could be applied to 445/615 (73%) of patients from the training set who had a median OS of 8.0 months (95% CI 6.7‐9.1). None of the patients from the external validation set were available owing to missing AST (Table S1). With the exception of risk group 2 vs 1 (HR 1.35, 0.94‐1.92, *P* = .102), there were clear survival differences between the four risk groups with a median OS ranging from 16.9 to 4.6 months (Figure 1) in risk groups 1 and 4, respectively.

### 3.3 Prognostic factors and improved model: PROSASH‐II

First, multiple imputation was performed on the training set to account for missing data (Table S1). An exploratory univariable analysis showed that albumin, Ln(bilirubin), ECOG PS, macrovascular invasion, extrahepatic spread, largest tumour size, number of liver lesions, Ln(AFP) and receiving prior HCC treatments were associated with OS (Table S2).

The stepwise multivariable regression identified albumin, Ln(bilirubin), macrovascular invasion, extrahepatic spread, largest tumour size and Ln(AFP) as statistically significant prognostic factors (Table 2). These six baseline variables and their coefficients were incorporated in a multivariable model, named the PROSASH‐II (Prediction Of Survival in Advanced Sorafenib‐treated HCC v2):

**Table 2:** Multivariable flexible parametric regression on imputed training set data

Variables	Hazard ratio (95% CI)	*P*‐value
Albumin – (g/L)	0.967 (0.951‐0.983)	<.001
Ln(Bilirubin) – µmol/L)	1.370 (1.178‐1.594)	<.001
Macrovascular invasion vs none	1.342 (1.124‐1.603)	.001
Extrahepatic spread vs none	1.198 (1.010‐1.420)	.038
Largest tumour size – cm	1.034 (1.016‐1.052)	<.001
LnAFP – U/L	1.073 (1.045‐1.101)	<.001
*Flexible parametric spline functions*		
γ_0_ (constant)	2.317 × 10^−2^ (0.916 × 10^−2^ to 5.858 × 10^−2^)	<.001
γ_1_	5.654 (4.274‐7.479)	<.001
γ_2_	1.034 (1.019‐1.050)	<.001

Note

Based on one interior knot with two degrees of freedom.

Abbreviations: 95% CI, 95% confidence interval; AFP, Alpha‐Foetoprotein; LN, natural logarithm.

**Table:** 

Linear predictor:	(−0.0337 × albumin in g/L)	+
	(0.315 × Ln(bilirubin in µmol/L))	+
	(0.295 × macrovascular invasion, where 0 = No and 1 = Yes)	+
	(0.181 × extrahepatic spread, where 0 = No and 1 = Yes)	+
	(0.0336 × Largest tumour size in cm)	+
	(0.0703 × Ln(AFP U/L))	

A comparison of the model variables using complete case data (Table S3) and imputed data showed very similar coefficients and *P* values, indicating that the model was not greatly impacted by the imputation of missing data.

Using the centile‐based cut‐points, four risk groups were created: ≤−0.0760 (risk group 1), >−0.0760 to ≤0.355 (risk group 2), >0.355 to ≤0.858 (risk group 3) and >0.858 (risk group 4).

To simplify individual survival prediction, the calculation for the linear predictor and risk groups was incorporated in an online calculator (https://jscalc.io/calc/qXgkZNB1h6B1jEfq). This calculator can be used to determine the risk group and chance of survival at 3, 6, 12 and 24 months for each patient. For example, a patient with an albumin of 45 g/L, a bilirubin of 7 µmol/L, an AFP of 5789 U/L, the largest tumour measuring 5.9 cm with macrovascular invasion, but without extrahepatic spread, will have a predicted survival of 87%, 70%, 44% and 19% and 9% at 3, 6, 12, 24 and 36 months, respectively. The equations for these predictions are detailed in Appendix S1.

### 3.4 PROSASH‐II performance in training and validation set

**Figure 2:**
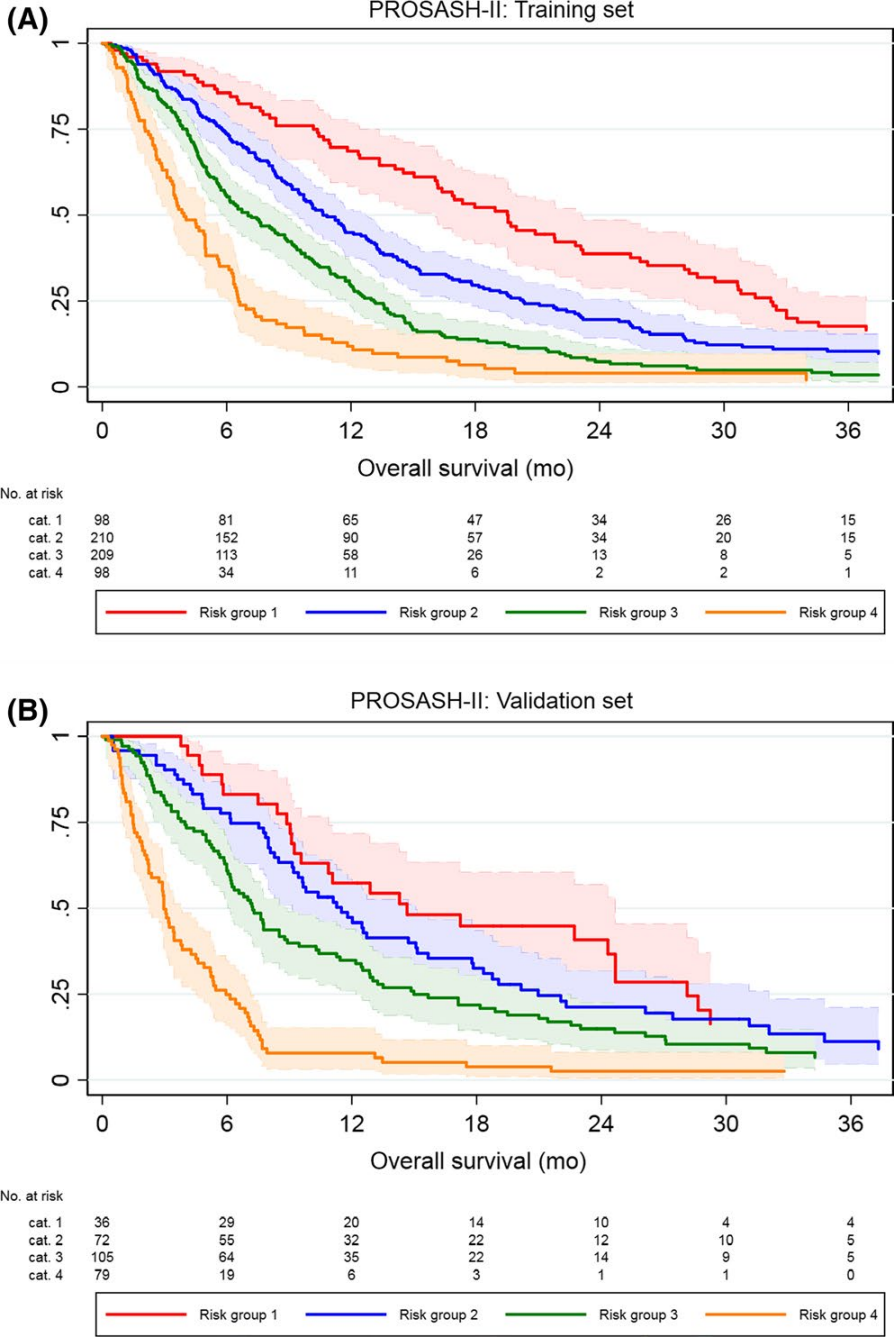
Overall survival according to the PROSASH‐II risk groups in the training (A) and validation (B) set with 95% confidence intervals

There were clear and statistically significant survival differences between the PROSASH‐II risk groups in the training set (Figure 2A), with a median OS ranging from 19.6 months (risk group 1) to 3.9 months (risk group 4). The PROSASH‐II model could be applied to 292 (93%) patients from the validation set. With the exception of risk group 1, which had fewer patients (n = 36, 12%) and showed overlap in 95% CI with risk group 2 (HR 1.32, 95% CI 0.85‐2.05, *P* = .220), the risk groups showed evenly good discrimination in the validation set (Figure 2B).

**Figure 3:**
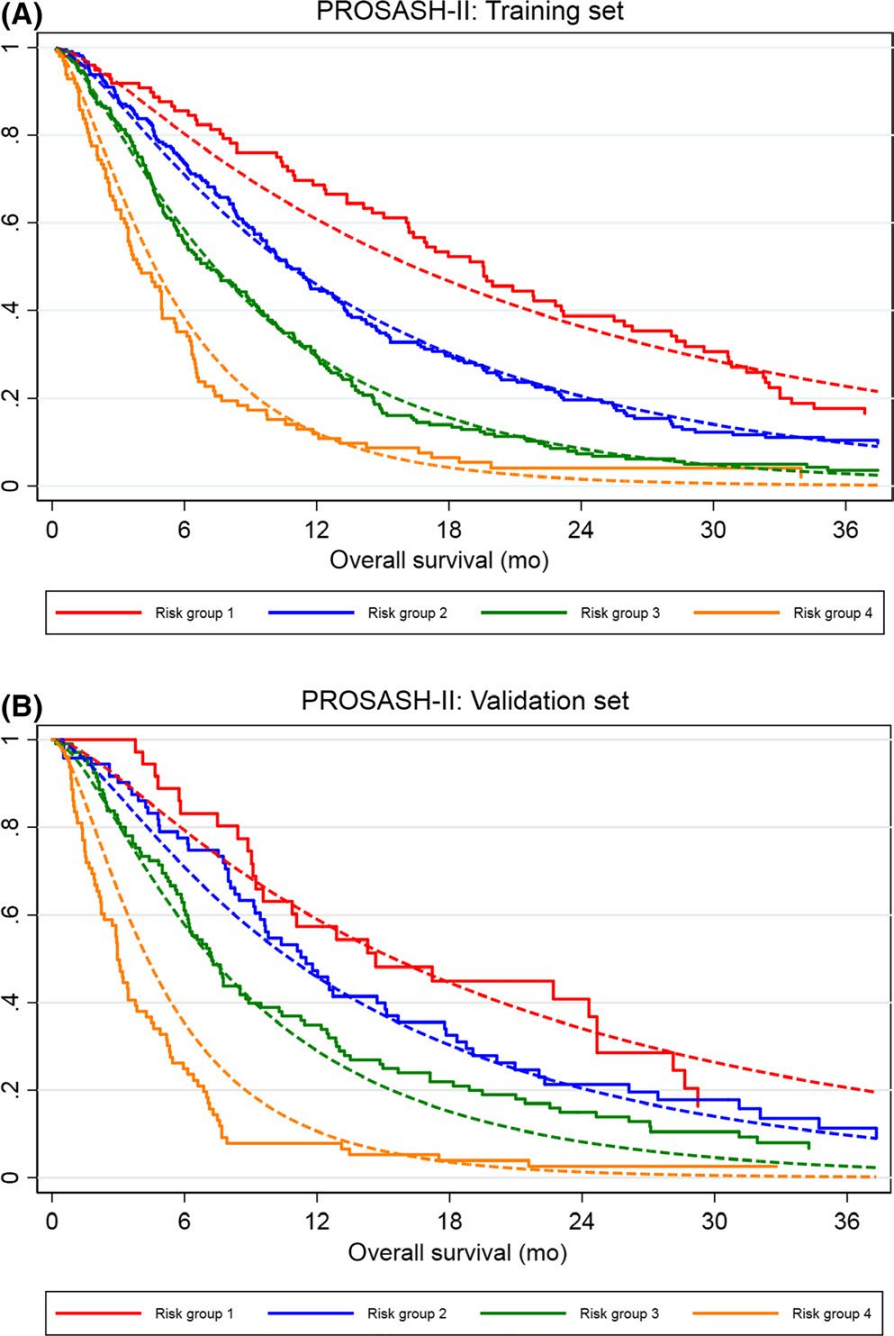
Calibration plot of the predicted (dotted line) and observed (solid line) of the overall survival according to PROSASH‐II risk groups in the training (A) and validation (B) set

Indicated by the concordance in the observed and predicted survival curves of both the training and validation sets (Figure 3A,B), the model showed good overall calibration. Similarly, the predicted and observed median OS and survival at 12 months closely matched in both datasets (Table 3). Although the model slightly underestimated the OS of risk group 1 in the training set, this was not the case in the validation set.

**Table 3:** Predicted vs observed survival of risk groups of the PROSASH‐II model

	Risk cat.	N	Observed mOS (95% CI)	Predicted mOS (95% CI)	Observed % survival at 12 mo. (95% CI)	Predicted % survival at 12 mo. (95% CI)	Hazard ratio (95% CI)	*P* value
Training (n = 615)	1	98	19.6 (16.1‐23.1)	16.4 (14.5‐19.5)	68.6 (58.3‐76.9)	61.3 (56.6‐66.4)	1	Reference
	2	210	10.6 (9.5‐12.7)	10.8 (9.8‐12.1)	45.0 (38.1‐51.7)	45.5 (41.8‐49.5)	1.49 (1.15‐1.93)	.003
	3	209	7.0 (5.9‐8.8)	7.5 (6.7‐8.1)	29.3 (23.2‐35.7)	29.3 (26.1‐32.9)	2.40 (1.85‐3.12)	<.001
	4	98	3.9 (3.3‐5.0)	4.7 (4.0‐5.6)	11.2 (6.4‐19.3)	14.4 (10.9‐18.9)	4.24 (3.13‐5.74)	<.001
Validation (n = 292)^a^	1	36	14.7 (9.2‐24.7)	16.6 (13.0‐18.5)	57.4 (39.5‐71.7)	58.3 (53.6‐63.4)	1	Reference
	2	72	11.5 (9.1‐15.1)	10.8 (9.8‐12.0)	47.3 (35.3‐58.4)	45.8 (42.0‐49.8)	1.32 (0.85‐2.05)	.220
	3	105	7.2 (6.0‐8.9)	7.2 (6.6‐7.8)	34.9 (25.9‐44.0)	28.8 (25.6‐32.4)	1.73 (1.14‐2.63)	.010
	4	79	3.0 (2.2‐3.8)	4.3 (3.8‐4.9)	7.9 (3.2‐15.2)	8.6 (6.1‐12.1)	4.84 (3.11‐7.54)	<.001
All (n = 907)	1	134	19.0 (14.7‐22.8)	16.2 (14.1‐19.0)	65.6 (56.8‐73.0)	60.8 (56.1‐65.9)	1	Reference
	2	282	11.2 (9.7‐12.5)	10.8 (9.8‐12.0)	45.6 (39.6‐51.4)	45.8 (42.1‐49.8)	1.44 (1.15‐1.80)	.001
	3	314	7.2 (6.2‐8.3)	7.3 (6.7‐8.1)	31.2 (26.1‐36.4)	29.4 (26.4‐33.0)	2.12 (1.70‐2.65)	<.001
	4	177	3.4 (3.0‐4.5)	4.5 (4.0‐5.2)	10.1 (5.1‐15.1)	11.2 (8.3‐15.3)	4.52 (3.54‐5.78)	<.001

Abbreviations: 95% CI, 95% confidence interval; mo, months; (m)OS, (median) overall survival; PROSASH, Prediction Of Survival in Advanced Sorafenib‐treated HCC.

a 13/305 patients could not be classified according to the PROSASH‐II model owing to missing values.

**Figure 4:**
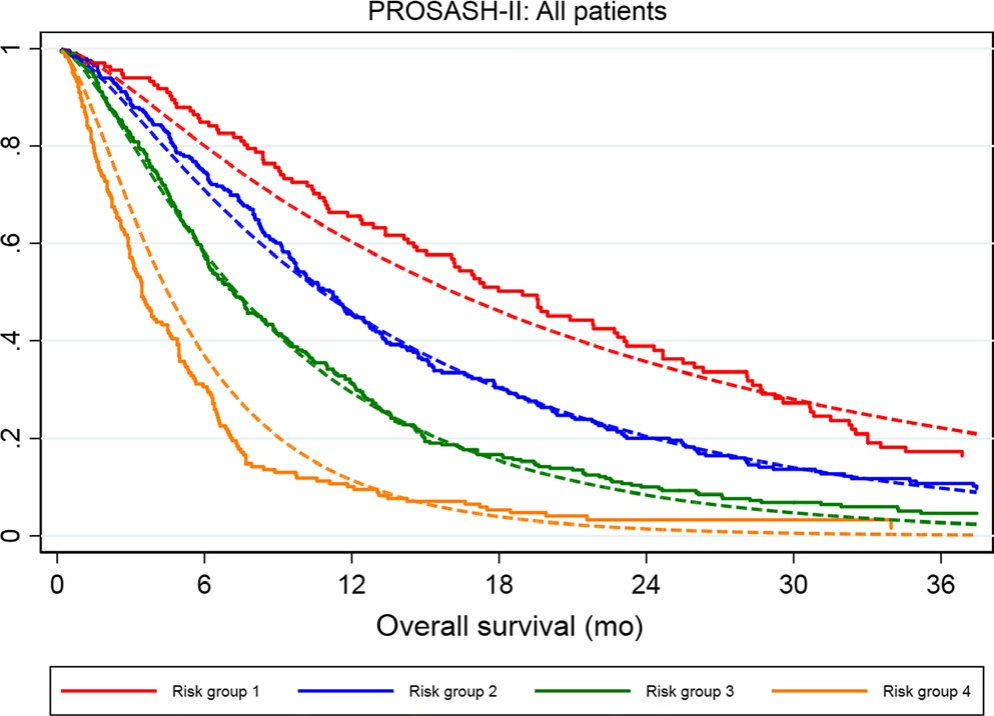
Calibration plot of the predicted (dotted line) and observed (solid line) of the overall survival according to the PROSASH‐II risk groups (1‐4) in all patients

Given the similarities of baseline characteristics and model performance in the training and validation sets, all patients were clustered together and then model‐based stratification was re‐applied. The median OS was 19.0, 11.2, 7.2 and 3.4 months with a 12‐month survival of 65.6%, 45.6%, 31.2% and 10.1%, in risk groups 1‐4, respectively. There was no overlap in hazard ratios (Table 3), indicating good discrimination. Similar to the training set, there was a trend towards a slight survival underestimation of patients in risk group 1 (Figure 4); however, overall, the predicted and observed survival were closely matched.

### 3.5 Subgroup analysis according to Child‐Pugh class

In a subgroup analysis of Child‐Pugh A patients (n = 767), who had a median OS of 9.1 months, there were clear survival differences between the various PROSASH‐II risk groups (Figure S2A). The median OS was 19.0, 10.8, 7.6 and 4.5 months across risk groups 1‐4, respectively. For the subgroup analysis of patients with Child‐Pugh B liver function, 136 patients were available with a median OS of 4.3 months (Figure S2B). None of these patients were assigned to risk group 1 and only 10 (13.4%) to risk group 2. There was a trend towards a poorer survival across risk groups 2 to 4 with a median OS of 13.4, 5.4 and 3.1 months, respectively. The difference between risk groups 2 and 3 was not significant owing to limited patient numbers (HR 1.98, 0.97‐4.04, *P* = .062). There were statistically significant survival differences between risk groups 3 and 4 (log‐rank *P* = .002).

### 3.6 PROSASH‐II model performance and comparison

The performance of the different prognostic models was compared and summarized in Tables 4 and 5. Comparisons were performed in the training set with imputed missing data (n = 615), the validation set with complete data (n = 290) and a subgroup of 438 patients with complete data for all prognostic models. Across the various prognostic models, there was a slight loss in discriminative power when patients were categorized in risk groups or prognostic classes. Moreover, there was a trend towards a higher C‐index and R^2^_D_ and lower AIC across all assessed prognostic models in the validation set compared with the training set. In all different subsets, the models with the lowest predictive performance in terms of AIC, C‐index and R^2^_D_ were the BCLC, Child‐Pugh and JIS. The HAP and SAP score performed very similarly in the different subsets.

**Table 4:** Comparison between of the predictive performance of prognostic models in the training and validation set

Staging system (no. of strata)	Imputed training set (n = 615)			Complete case validation set (n = 290)		
	AIC	C‐index (IQR^b^)	R^2^_D_ (95% CI^a^)	AIC	C‐index (95% CI^a^)	R^2^_D_ (95% CI^a^)
PROSASH‐II						
Linear predictor	1684	0.65 (0.64‐0.65)	0.12 (0.08‐0.17)	828	0.68 (0.65‐0.72)	0.16 (0.08‐0.24)
Grouped (4)	1697	0.64 (0.64‐0.64)	0.12 (0.08‐0.17)	839	0.67 (0.64‐0.70)	0.16 (0.09‐0.25)
PROSASH						
Linear predictor	—	—	—	—	—	—
Grouped (4)	—	—	—	—	—	—
ALBI						
Linear predictor	1764	0.59 (0.59‐0.59)	0.04 (0.01‐0.06)	867	0.62 (0.58‐0.65)	0.06 (0.03‐0.13)
Grade (3)	1781	0.56 (0.55‐0.56)	0.03 (<0.01‐0.05)	877	0.58 (0.55‐0.61)	0.05 (0.01‐0.12)
Child‐Pugh (3)	1782	0.53 (0.53‐0.53)	0.05 (0.01‐0.09)	867	0.58 (0.55‐0.61)	0.11 (0.04‐0.21)
BCLC (4)^c^	1785	0.54 (0.52‐0.56)	0.02 (<0.01‐0.06)	885	0.57 (0.55‐0.60)	0.03 (0.01‐0.08)
HAP						
Points (5)	1733	0.60 (0.60‐0.60)	0.08 (0.04‐0.12)	833	0.67 (0.64‐0.70)	0.16 (0.09‐0.25)
Classes (4)	1738	0.60 (0.60‐0.60)	0.08 (0.04‐0.11)	840	0.66 (0.63‐0.69)	0.14 (0.07‐0.23)
SAP						
Points (5)	1733	0.60 (0.60‐0.61)	0.08 (0.04‐0.12)	817	0.69 (0.66‐0.72)	0.16 (0.09‐0.27)
Classes (3)	1738	0.59 (0.59‐0.59)	0.09 (0.04‐0.13)	830	0.66 (0.63‐0.69)	0.14 (0.07‐0.23)
JIS (5)	1775	0.55 (0.55‐0.55)	0.03 (0.01‐0.06)	877	0.59 (0.55‐0.62)	0.05 (0.02‐0.12)

Abbreviations: 95% CI, 95% confidence interval; AIC, Akaike Information Criterion; ALBI; albumin‐bilirubin; C‐index, BCLC, Barcelona Clinic Liver Cancer; HAP, Hepatoma Arterial‐embolization Prognostic score; Harrell's C‐index; JIS, Japan Integrated Staging score; R^2^D, Royston‐Sauerbrei's R^2^D; PROSASH, Prediction Of Survival in Advanced Sorafenib‐treated HCC; SAP, Sorafenib Advanced HCC Prognostic score.

a Confidence intervals estimated from 200 bootstrap samples.

b Median and IQR for each model were estimated from the 10 imputed linear predictors.

c Only n = 1 missing in training cohort, thus a complete case analysis was performed.

**Table 5:** Comparison of prognostic models in a complete case population

Staging system (no. of strata)	Complete case for all models (n = 438)		
	AIC	C‐index (95% CI*)	R^2^_D_ (95% CI^a^)
PROSASH‐II			
Linear predictor	1260	0.63 (0.60‐0.66)	0.10 (0.06‐0.15)
Grouped (4)	1266	0.62 (0.60‐0.65)	0.10 (0.05‐0.15)
PROSASH			
Linear predictor	1278	0.62 (0.59‐0.65)	0.07 (0.04‐0.11)
Grouped (4)	1279	0.61 (0.58‐0.64)	0.08 (0.04‐013)
ALBI			
Linear predictor	1303	0.58 (0.55‐0.61)	0.03 (0.01‐0.07)
Grade (3)	1318	0.54 (0.52‐0.57)	0.02 (<0.01‐0.05)
Child‐Pugh (3)	1317	0.52 (0.51‐0.54)	0.04 (0.01‐0.07)
BCLC (4)	1320	0.53 (0.51‐0.56)	0.01 (<0.01‐0.04)
HAP			
Points (5)	1289	0.59 (0.56‐0.62)	0.06 (0.03‐0.11)
Classes (4)	1292	0.59 (0.56‐0.62)	0.06 (0.03‐0.11)
SAP			
Points (5)	1293	0.59 (0.56‐0.62)	0.05 (0.02‐0.09)
Classes (3)	1291	0.58 (0.55‐0.61)	0.07 (0.03‐0.13)
JIS (5)	1315	0.53 (0.51‐0.56)	0.02 (<0.01‐0.05)

Abbreviations: 95% CI, 95% confidence interval; AIC, Akaike Information Criterion; ALBI; albumin‐bilirubin; C‐index, BCLC, Barcelona Clinic Liver Cancer; HAP, Hepatoma Arterial‐embolization Prognostic score; Harrell's C‐index; JIS, Japan Integrated Staging score; R^2^D, Royston‐Sauerbrei's R^2^D; PROSASH, Prediction Of Survival in Advanced Sorafenib‐treated HCC; SAP, Sorafenib Advanced HCC Prognostic score.

a Confidence intervals estimated from 200 bootstrap samples.

In the training set, the higher C‐index (0.65, IQR 0.64‐0.65) and R^2^_D_ (0.12, 95% CI 0.08‐0.17) of the PROSASH‐II indicated improved discriminative performance and explained variation compared with the currently available models. Likewise, the PROSASH‐II had a lower AIC (1684) which indicated a better goodness of fit.

In the validation set, the PROSASH‐II model had a higher C‐index (0.68, 95% CI 0.65‐0.72) and lower AIC (828) than commonly used scores such as BCLC and Child‐Pugh. It also had the highest R^2^_D_ (0.16, 95% CI 0.08‐0.24) of all tested models, reflecting better explained variation. However, the model appeared to have a similar prognostic performance as the HAP and SAP scores, the latter showing a slightly higher C‐index (0.69, 95% CI 0.66‐0.72) and lower AIC (817) than the PROSASH‐II model.

In the complete case subset for all models (n = 438), the PROSASH‐II model had the highest C‐index (0.63, 95% CI 0.60‐0.66) and R^2^_D_ (0.10, 95% CI 0.06‐0.15) and lowest AIC (1260). The slightly poorer values for AIC (1278), C‐index (0.62, 95% CI 0.59‐0.65) and R^2^_D_ (0.07, 95% CI 0.04‐0.11) of the original PROSASH model indicated a comparable predictive performance.

## 4 DISCUSSION

In this large multicentre study of patients treated with sorafenib for HCC, the clinical trial‐based PROSASH model was successfully validated and optimized (PROSASH‐II) in routine clinical practice. The PROSASH‐II model, which uses fewer and more objective parameters and performed at least as good as PROSASH, offers individualized survival prediction and performs better than frequently used prognostic models (ie BCLC and Child‐Pugh).

In light of the modest survival benefit (2‐3 months) and significant costs and toxicity of sorafenib in advanced HCC, various studies have raised concerns on the cost‐effectiveness of sorafenib in daily practice.^42^, ^43^, ^44^ The BCLC staging system and Child‐Pugh score are the most used prognostic models, but they have clear limitations: Child‐Pugh incorporates subjective parameters which can lead to misclassification and inter‐observer variability,^9^ whereas the prognostic value of BCLC staging for patients treated with the same modality is low. To optimize cost‐effectiveness and aid clinicians in survival prediction and clinical decision‐making, several other prognostic models have been proposed to stratify these patients (Table 6). Interestingly, most of these models were not specifically built for sorafenib‐treated HCC patients and none of them performed optimal.^9^, ^18^, ^19^, ^45^, ^46^, ^47^ Lack of consensus, easy applicability and external validation have hampered implementation of these prognostic scores in clinical practice.

**Table 6:** Literature reported performance of prognostic models patients with HCC treated with sorafenib

Name model	Variables			C‐index	Type of cohort (n)	References
	Tumour‐related	Liver function	Other			
*Tested in this study*						
PROSASH‐II	AFP EHS MVI Tumour size	Bilirubin Albumin		0.65 0.68	Training (615) Validation (290)	Present study Present study
PROSASH	AFP EHS MVI	AST Albumin	Aetiology Age Creatinine	0.72 0.70 0.62	Training (500) Validation (421) Validation (438)	Berhane et al^21^ Berhane et al^21^ Present study
ALBI		Albumin Bilirubin		0.60 0.60 NA 0.59 0.62	Validation (905) Validation (468) Validation (681) Validation (615) Validation (290)	Edeline et al^9^ Edeline et al^18^ Samawi et al^46^ Present study Present study
Child‐Pugh		Albumin Bilirubin PT/INR Ascites Encephalopathy		0.61 0.53 0.58	Validation (905) Validation (615) Validation (290)	Edeline et al^9^ Present study Present study
BCLC	ECOG PS EHS MVI	Child‐Pugh		0.64 0.55 NA 0.54 0.57	Validation (435) Validation (468) Validation (681) Validation (615) Validation (290)	Takeda et al^19^ Edeline et al^18^ Samawi et al^46^ Present study Present study
HAP	AFP Tumour size	Albumin Bilirubin		0.65 0.60 0.67	Validation (468) Validation (615) Validation (290)	Edeline et al^18^ Present study Present study
SAP	ECOG PS AFP Tumour size	Albumin Bilirubin		0.64 0.60 0.69	Validation (468) Validation (615) Validation (290)	Edeline et al^18^ Present study Present study
JIS	Tumour size Tumour number MVI	Child‐Pugh		0.69 0.55 0.59	Validation (435) Validation (615) Validation (290)	Takeda et al^19^ Present study Present study
*Not tested in this study*						
CLIP	AFP MVI Tumour number % Tumour extent	Child‐Pugh		0.54 NA	Validation (435) Validation (681)	Takeda et al^19^ Samawi et al^46^
Okuda	% Tumour extent	Albumin Bilirubin Ascites		0.63 NA	Validation (435) Validation (681)	Takeda et al^19^ Samawi et al^46^
JRC	AFP DCP EHS MVI Morphology	Albumin Bilirubin		0.76	Training (435)	Takeda et al^19^
NIACE	ECOG PS AFP Morphology Tumour number	Child‐Pugh		NA	Validation (83) Validation (83) Validation (119)	Adhoute et al^17^
AJCC TNM7	Tumour size Tumour number MVI EHS			NA	Validation (681)	Samawi et al^46^
Korean	AFP Morphology MVI	Child‐Pugh		NA	Training (612)	Yoo et al^20^

Abbreviations: AFP, Alpha‐Foetoprotein; AJCC TNM, American Joint Committee on Cancer Tumour Node Metastasis; ALBI; albumin‐bilirubin; AST, aspartate transaminase; BCLC, Barcelona Clinic Liver Cancer; CLIP, Cancer of the Liver Italian Program score; DPC, Des‐gamma‐carboxy prothrombin; ECOG PS, Eastern Cooperative Oncology Group performance status; EHS, extrahepatic spread; HAP, hepatoma arterial‐embolization prognostic score; JIS, Japan Integrated Staging score; JRC, Japan Red Cross score; MVI. Macrovascular invasion; PROSASH, Prediction Of Survival in Advanced Sorafenib‐treated HCC; SAP, Sorafenib Advanced HCC Prognostic score.

We were able to compare eight different prognostic models: ALBI, Child‐Pugh, BCLC, HAP, SAP, JIS, PROSASH and the newly proposed PROSASH‐II model (Table 6). All tested models included parameters for liver function (ie albumin, bilirubin, AST), most of them included tumour‐related parameters (ie AFP, tumour size, macrovascular invasion) and some included ‘other’ baseline parameters (age, HCC aetiology, ECOG PS). Only a few scores have incorporated *predictive* parameters that were associated with increased benefit of sorafenib over placebo (extrahepatic spread, NLR and hepatitis C virus infection).^16^ This may reflect the modest impact of sorafenib on the natural history of advanced HCC. The well‐known prognostic impact of the severity of the underlying liver disease was confirmed in this study, reflected by multivariable significance and incorporation of albumin in the PROSASH and PROSASH‐II models. In accordance with prior studies,^9^, ^48^, ^49^ we showed that despite using less parameters, ALBI has a better discrimination than the Child‐Pugh classification.

Although initially developed to stratify HCC patients treated with TACE, the HAP score showed that a further improvement of predictive accuracy is possible by combining liver function (albumin, bilirubin) and tumour‐related (AFP, tumour size) parameters.^18^ The highly comparable SAP score, which adds ECOG PS, performed similarly in our study. Depending on the subgroup of patients, the HAP and SAP scores performed slightly worse or similar to the PROSASH and PROSASH‐II models. Given the overlap of four prognostic parameters (albumin, bilirubin, AFP and tumour size) which are dichotomized in the HAP and SAP scores, this is not unexpected. However, neither the SAP nor HAP score offer individualized survival prediction and do not incorporate predictors of improved sorafenib benefit.

Both the PROSASH and PROSASH‐II models offer individualized survival prediction and propose an externally validated four‐tier subgroup classification with a median survival of 17‐10‐7‐5 months and 19‐11‐7‐3 months, for risk groups 1‐4, respectively. The PROSASH incorporated albumin, AFP, AST, creatinine, age, extrahepatic spread, macrovascular invasion, ECOG PS and disease aetiology (nine parameters in total), whereas the PROSASH‐II incorporated albumin, AFP, extrahepatic spread, macrovascular invasion, tumour size and bilirubin (six parameters in total). It is inevitable that different studies with different datasets lead to (slightly) different prognostic models. However, despite the different origins (clinical trial vs daily practice), there is significant overlap in the PROSASH‐I and ‐II variables which suggests that these variables are stable and clinically relevant. As pointed out by several statistical experts, there is no widely agreed approach to build a multivariable prognostic model from a set of candidate predictors.^50^, ^51^ In this study, we aimed to report on the optimized statistical associations in daily clinical practice of sorafenib‐treated patients guided by two main principles in prognostic model building. Firstly, the parameters should be commonly available in centres treating patients with HCC. Secondly, models should be widely validated and universally applicable. For this purpose, we used large international datasets that have inevitable differences in data availability. As suggested by Royston et al, this was handled by multiple imputation of randomly missing data (Table S1) and by balancing data availability (ie parameter selection) and analytic power (ie patient numbers).^50^ Using this approach, we were able to build the PROSASH‐II model which required fewer and only highly reproducible parameters while it performed better in terms of C‐index, AIC and R^2^_D_ than its predecessor. Disease aetiology and ECOG PS are less objective parameters which may lead to inter‐ and intra‐user variability in daily practice, favouring PROSASH‐II as a tool that can aid clinicians in providing patient‐tailored treatment. Moreover, PROSASH‐II was built and tested on a daily clinical practice population in which it will be applied. Currently, guidelines recommend to consider all patients with well‐preserved liver function (Child‐Pugh‐A) who are unsuitable for loco‐regional therapy for sorafenib treatment. The clear subgroup survival differences of PROSASH‐II risk groups in Child‐Pugh A patients show that even in ‘guideline concordant patients’ a more individualized decision is possible. Patients within risk group 3 (median OS 7‐8 months) may have more benefit from alternative treatments (lenvantinib, clinical trials ie with PD1/PD‐L1 blockers), whereas patients within risk group 4 (median OS 3‐5 months) could be counselled to receive best‐supportive care only. A similar stratification was seen in patients classified as Child‐Pugh B who are currently not recommended to be treated with sorafenib and have a poor prognosis (median OS of 4.3 months). Still, a small subgroup of these patients (risk group 2, <10%) had a better prognosis (risk group 2, median OS 13.4 months) and could be considered for treatment with sorafenib.

In addition, the PROSASH‐II stratification could be used for pre‐planned or post‐hoc subgroup analyses of ongoing and finalized phase‐III studies comparing sorafenib with alternative treatments. Another application would be to generate survival curves of patients with advanced HCC treated with new agents in phases I‐II studies. A quantitative comparison between the observed survival outcomes of tested agent and the predicted sorafenib survival remains difficult in these ‘in silico’ clinical trials, but it could aid in deciding whether these agents can proceed to be tested in a phase III trial.

This study has several limitations, foremost the retrospective design and its inherent limitations. Owing to missing parameters, some previously proposed prognostic factors (ie NLR,^16^, ^52^, ^53^, ^54^, ^55^ body composition^13^, ^56^) could not be taken into account and not all previously proposed models could be included in the comparison (CLIP, NIACE). Secondly, this study was performed in patients treated in European countries and should be validated in other geographical areas (i.e. Asia).

Despite over a decade of sorafenib usage and extensive studies, no molecular markers with a strong association with mechanism of sorafenib action have been identified, reflecting the complexity of advanced stage HCC and the difficulty of simplifying this into easily applicable biomarkers.^8^ Our calculator provides a clinically applicable and validated model for the unmet need of outcome prediction prior to sorafenib treatment. Future studies could improve the risk stratification, survival prediction and clinical decision‐making by not only taking into account baseline factors (pre‐sorafenib) but also parameters that can be monitored and may be of potential prognostic influence during treatment (ie sorafenib dose, dynamics in liver function, AFP, radiological response or pattern of progression). The more recently approved second‐line treatments for advanced HCC (ie regorafenib [2017], cabozantinib [2019]) most likely did not have a major impact on the current model because the included patients were treated with sorafenib prior to FDA/EMEA approval of these treatments and the landmark trials of these agents had strict inclusion criteria. Future studies aiming to implement these variables into robust tools and validated models will require large collaborations with detailed and high‐quality (prospective) datasets. To avoid statistical bias (overfitting), it remains important to externally validate novel prognostic models.

In conclusion, our study validated the PROSASH model in routine daily practice and proposed an improved model (PROSASH‐II) which uses less and more objective clinical features. The PROSASH‐II model outperforms the currently available models and offers risk group stratification and individualized survival prediction that can be used for tailored treatment of HCC in daily practice and pre‐planned subgroups analyses of future studies.

## CONFLICTS OF INTEREST

DB receives teaching and speaking fees from Bayer Healthcare and from the Falk Foundation, Germany. HJK is a member of the advisory board for Ipsen and Sirtex. JE receives speaking fees and travel grants from Bayer. JFB is a member of the advisory board for Bayer, IPSEN, ESAI. RBT served as a speaker for Gore WL, Bayer and Norgine. He is a member of the advisory board for Gilead and Norgine. RdM served as a speaker for Norgine and as a consultant for Cook Medical. OvD served as a consultant for Cook Medical. All other authors have declared no conflicts of interest. This study was designed and conducted by academic investigators.

## AUTHORS’ CONTRIBUTIONS

TL lead this study, performed data acquisition and analysis, and wrote the manuscript. SB and LB performed and supervised data analysis. JE, JFB, DB, TM, JvV, DtC, RdM, FE, AC, OvD, HJK and RBT supervised this study, provided data and clinical input and provided mentorship for this study. PJ conceived this study design and is the guarantor of the article. All authors have reviewed and approved a final version of the manuscript.
