# Supplementary material for: Improved survival prediction and comparison of prognostic models for patients with hepatocellular carcinoma treated with sorafenib
Source: Liver Int. 2019 Nov 18;40(1):215–28. doi: 10.1111/liv.14270 (PMC6973249; doi:10.1111/liv.14270)
Supplement: Supplementary file 1 [file LIV-40-215-s001.docx]

**Supplementary Tables and Graphs**

| **Supplementary Table 1.** Number of missing data in the baseline parameters. | | | |
| --- | --- | --- | --- |
| **Variables (%)** | **Entire cohort** | **Training-set** | **External validation** |
|  | n=920 | n=615 | n=305 |
| **Demographics** |  |  |  |
| **Age** | 0 (0) | 0 (0) | 0 (0) |
| **Sex** | 0 (0) | 0 (0) | 0 (0) |
| **Liver disease** |  |  |  |
| **Aetiology** | 0 (0) | 0 (0) | 0 (0) |
| **Child-Pugh class** | 36 (4) | 35 (6) | 1 (<1) |
| **Tumour parameters** |  |  |  |
| **ECOG PS** | 0 (0) | 0 (0) | 0 (0) |
| **Number of liver lesions** | 18 (2) | 6 (1) | 12 (4) |
| **Largest tumour size** | 22 (2) | 13 (2) | 9 (3) |
| **Macrovascular invasion** | 1 (<1) | 1 (<1) | 0 (0) |
| **Extra-hepatic spread** | 2 (<1) | 1 (<1) | 1 (<1) |
| **BCLC stage** | 1 (<1) | 1 (<1) | 0 (0) |
| **Prior treatments** | 0 (0) | 0 (0) | 0 (0) |
| **Serum tests** |  |  |  |
| **AFP** | 26 (3) | 25 (4) | 1 (<1) |
| **Albumin** | 61 (7) | 59 (10) | 2 (1) |
| **Bilirubin** | 30 (3) | 29 (5) | 1 (<1) |
| **AST*** | 449 (49) | 144 (23) | 305 (100) |
| **Creatinine** | 69 (8) | 35 (6) | 34 (11) |
| **Survival outcomes** |  |  |  |
| **Death** | 0 (0) | 0 (0) | 0 (0) |
| **Median overall survival** | 0 (0) | 0 (0) | 0 (0) |
| *Was not included in multiple imputation as values were not missing at random in external validation set.  Abbreviations: AFP, Alpha-Fetoprotein; AST, aspartate transaminase; BCLC, Barcelona Clinic Liver Cancer; ECOG PS, Eastern Cooperative Oncology Group performance status. | | | |

| **Supplementary Table 2.** Univariable flexible parametric regression on the imputed training set data (n=615) | | |
| --- | --- | --- |
| **Variables used in model building** | **Hazard ratio (95% CI)** | ***p*-value*** |
| Age – (years) | 0.993 (0.986-1.001) | 0.106 |
| Male gender – versus female | 0.940 (0.753-1.173) | 0.585 |
| HBV – versus none | 1.179 (0.919-1.512) | 0.196 |
| HCV – versus none | 0.946 (0.745-1.202) | 0.651 |
| Alcohol – versus none | 1.100 (0.925-1.310) | 0.281 |
| Albumin – (g/l) | 0.961 (0.946-0.977) | <0.001 |
| Ln(Bilirubin) – µmol/l) | 1.417 (1.228-1.636) | <0.001 |
| ECOG PS |  |  |
| 0 | Reference | - |
| 1 | 1.180 (0.993-1.402) | 0.060 |
| 2 | 1.530 (1.099-2.130) | 0.012 |
| Macrovascular invasion – versus none | 1.465 (1.123-1.741) | <0.001 |
| Extrahepatic spread – versus none | 1.183 (1.002-1.400) | 0.047 |
| Largest tumour size – cm | 1.041 (1.023-1.059) | <0.001 |
| Number of liver lesions |  |  |
| 1 | Reference | - |
| 2-3 | 0.784 (0.618-0.995) | 0.046 |
| >3 | 0.932 (0.755-1.151) | 0.514 |
| LnAFP – U/L | 1.091 (1.064-1.119) | <0.001 |
| lnCreatinine – µmol/l | 0.988 (0.713-1.370) | 0.944 |
| Sorafenib was initial treatment – versus prior other | 1.328 (1.124-1.568) | 0.001 |
| Abbreviations: 95% CI, 95% confidence interval; AFP, Alpha-Fetoprotein; BCLC, Barcelona Clinic Liver Cancer; ECOG PS, Eastern Cooperative Oncology Group performance status; HBV, hepatitis B virus; HCV, hepatitis C virus. | | |

| **Supplementary Table 3.** Comparison of multivariable flexible parametric regression in the training set with complete or imputed data | | | | |
| --- | --- | --- | --- | --- |
|  | ***Complete case (n=529)*** | | ***Imputed data (n=615)*** | |
| **Variables** | **Hazard ratio (95% CI)** | ***p*-value** | **Hazard ratio (95% CI)** | ***p*-value** |
| Albumin – (g/l) | 0.969 (0.952-0.986) | <0.001 | 0.967 (0.951-0.983) | <0.001 |
| Ln(Bilirubin) – µmol/l) | 1.403 (1.193-1.649) | <0.001 | 1.370 (1.178-1.594) | <0.001 |
| Macrovascular invasion – versus none | 1.301 (1.077-1.572) | 0.006 | 1.342 (1.124-1.603) | 0.001 |
| Extrahepatic spread – versus none | 1.193 (0.994-1.432) | 0.058 | 1.198 (1.010-1.420) | 0.038 |
| Largest tumour size – cm | 1.030 (1.012-1.050) | 0.001 | 1.034 (1.016-1.052) | <0.001 |
| LnAFP – U/L | 1.073 (1.043-1.103) | <0.001 | 1.073 (1.045-1.101) | <0.001 |
| Abbreviations: 95% CI, 95% confidence interval; AFP, Alpha-Fetoprotein. | | | | |

**Supplementary Figure 1**. Overall survival according to training or validation set.

| **Group** | **N** | **Median OS, months (95% CI)** | **Hazard ratio (95% CI)** | ***p*-value** |
| --- | --- | --- | --- | --- |
| Training | 615 | 8.9 (8.0-9.8) | 1 | Reference |
| Validation | 305 | 7.7 (6.8-8.8) | 1.05 (0.91-1.21) | 0.128 |

| **Group (Child-Pugh A)** | **N** | **Median OS, months (95% CI)** | **Hazard ratio (95% CI)** | ***p*-value** |
| --- | --- | --- | --- | --- |
| Risk group 1 | 134 | 19.0 (14.7-22.8) | 1 | Reference |
| Risk group 2 | 272 | 10.8 (9.6-12.5) | 1.45 (1.16-1.81) | 0.001 |
| Risk group 3 | 258 | 7.6 (6.3-8.8) | 2.05 (1.63-2.57) | <0.001 |
| Risk group 4 | 103 | 4.5 (3.3-5.0) | 4.31 (3.27-5.69) | <0.001 |

| **Group (Child-Pugh B)** | **N** | **Median OS, months (95% CI)** | **Hazard ratio (95% CI)** | ***p*-value** |
| --- | --- | --- | --- | --- |
| Risk group 1 | 0 | - | - | - |
| Risk group 2 | 10 | 13.4 (1.2-24.9) | 1 | Reference |
| Risk group 3 | 55 | 5.4 (4.1-8.3) | 1.98 (0.97-4.04) | 0.062 |
| Risk group 4 | 71 | 3.1 (2.1-3.8) | 3.63 (1.76-7.50) | 0.001 |

**Supplementary Figure 2A and 2B**. Overall survival according to the PROSASH-II risk categories in patients classified as Child-Pugh A and Child-Pugh B.

**Supplementary Appendix A**

Survival function, S(t), at time t for an individual subject can then be defined as:

S(t) = S0(t)exp(η)

where S0(t) is the baseline survival function = exp(-exp(s(log t | γ))) and η is the linear predictor of the model.

To derive the log cumulative baseline hazard (spline function) at time t:

s(log t *| γ*) = γ_0_ + γ_1_z_1_ + γ_2_z_2_

(1) The log cumulative baseline hazard (spline function) at time t (in months) was derived as follows:

s(log t) = -3.765 + (1.732 * log t) + (0.034 * z_2_)

where z_2_ = ${(log t - 2.077)}_{+}^{3}$ – $0.348{(log t+1.806)}_{+}^{3}$ – $0.652{(log t - 4.148)}_{+}^{3}$

The “${(\ldots)}_{+}^{3}$” notation denotes (x)_+_=max{0, x}. This means that in case of negative value between the brackets, this is replaced by 0.

(2) Baseline survival function, S_0_(t), at time t was expressed as:

S_0_(t) = exp(-exp(s(log t)))

(3) Survival function, S(t), which gives the probability of an individual patient to survive beyond time t can then be calculated by:

S(t) = S_0_(t)^exp(η)^

where η is the linear predictor.

The values for S_0_(t) at time points 3, 6, 12, 24 and 36 months were 0.890, 0.743, 0.509, 0.252 and 0.132 respectively. For other time points, S_0_(t) can be calculated by following Steps 1 and 2.
